# Kinetic Modeling of Sunflower Grain Filling and Fatty Acid Biosynthesis

**DOI:** 10.3389/fpls.2016.00586

**Published:** 2016-05-06

**Authors:** Ignacio Durruty, Luis A. N. Aguirrezábal, María M. Echarte

**Affiliations:** ^1^Grupo de Ingeniería Bioquímica, CONICET, Departamento de Ingeniería Química y de Alimentos, Fac. Ingeniería, Universidad Nacional de Mar del PlataMar del Plata, Argentina; ^2^Laboratorio de Fisiología Vegetal, CONICET, Unidad Integrada Balcarce (Instituto Nacional de Tecnología Agropecuaria-Facultad de Ciencias Agrarias, Universidad Nacional de Mar del Plata)Balcarce, Argentina

**Keywords:** sunflower, grain filling, fatty acids biosynthesis, kinetic model

## Abstract

Grain growth and oil biosynthesis are complex processes that involve various enzymes placed in different sub-cellular compartments of the grain. In order to understand the mechanisms controlling grain weight and composition, we need mathematical models capable of simulating the dynamic behavior of the main components of the grain during the grain filling stage. In this paper, we present a non-structured mechanistic kinetic model developed for sunflower grains. The model was first calibrated for sunflower hybrid ACA855. The calibrated model was able to predict the theoretical amount of carbohydrate equivalents allocated to the grain, grain growth and the dynamics of the oil and non-oil fraction, while considering maintenance requirements and leaf senescence. Incorporating into the model the serial-parallel nature of fatty acid biosynthesis permitted a good representation of the kinetics of palmitic, stearic, oleic, and linoleic acids production. A sensitivity analysis showed that the relative influence of input parameters changed along grain development. Grain growth was mostly affected by the specific growth parameter (μ′) while fatty acid composition strongly depended on their own maximum specific rate parameters. The model was successfully applied to two additional hybrids (MG2 and DK3820). The proposed model can be the first building block toward the development of a more sophisticated model, capable of predicting the effects of environmental conditions on grain weight and composition, in a comprehensive and quantitative way.

## Introduction

The weight and composition of oilseed grains at harvest are complex traits that depend on the dynamics of many processes occurring earlier at both the plant and organ levels. Their response to the genotype and the environment results from several linked processes controlled at different levels of organization, from sub-cellular to crop (Martre et al., 2011). The sensitivity of these traits to multiple factors changes during grain development (Aguirrezábal et al., 2003; Rondanini et al., 2003; Echarte et al., 2013). Therefore, understanding how grain weight and composition are determined demands a deep and integrated knowledge of grain filling dynamics.

Most of the photoassimilates supplied to the sunflower grains during their filling period are contemporaneously synthesized by the leaves and thus, leaves are considered the main source of substrate for grain growth (Hall et al., 1990; López Pereira et al., 2008; Echarte et al., 2012). Since grains are the main sink of photoassimilates during this period, changes in assimilate production at the source can be interpreted as changes in carbon availability in the developing grains (Hall et al., 1995), sucrose being the major carbohydrate and the main phloem-transported sugar in sunflower plants (Alkio et al., 2002). Once in the grain, part of this carbon is directed to the pool of acetyl-CoA, the precursor of fatty acids, which are the main components of sunflower oil.

The biosynthesis of fatty acids involves several enzymes placed in different sub-cellular compartments of the grains (Garcés and Mancha, 1991; Gray and Kekwick, 1996; Harwood, 1996). The enzyme acetyl-CoA carboxylase (ACCase) catalyzes the first reaction of this pathway. After successive elongation reactions, the main products of the intraplastidial *de novo* fatty acid biosynthesis are palmitoyl-ACP (Pp), stearoyl-ACP (Sp), and oleoyl-ACP (Op). These acyl-ACPs are hydrolyzed to free fatty acids, activated to the corresponding acyl-CoAs, and exported to the cytosol to be incorporated into glycerolipids. Finally, oleic acid can be transformed into linoleic acid by the action of oleoylphosphatidylcholine desaturase (FAD2), an enzyme located in the endoplasmic reticulum (E.R), in a step that represents a key point in the regulation of fatty acid composition (Garcés and Mancha, 1991).

Progress in understanding and modeling grain weight and composition dynamics can be achieved with two different main approaches: empirical or mechanistic. While empirical models typically describe the data but do not explain them, mechanistic models are reductionist and explain data based on knowledge of processes at the lower levels of biological organization (Loomis et al., 1979; Thornley and Johnson, 1990). Mechanistic models of physiological processes are often based on biochemical principles such as enzyme kinetics and reaction stoichiometry (Amthor, 2000). For instance, a biochemical model of fatty acids biosynthesis has been proposed by Martínez-Force and Garcés (2002). This model is a structured kinetic one based on every known enzymatic step of the pathway. Although useful to understand the fatty acid biosynthetic pathway, this model does not consider the supply of substrate by the mother plant (which changes along grain filling), nor the synthesis of other grain components that contribute to grain weight and composition. The formulations of this kind of model require extensive knowledge of metabolic pathways, often not available in enough detail, and complex computer programming to carry out the calculations involved (Martínez-Force and Garcés, 2002). Furthermore, the concentrations of intermediate compounds and enzymatic activities are often difficult to measure, hindering validation of state variables, and making them less attractive for practical applications (Durruty et al., 2012).

Mathematical models with a more empirical approach have been developed to predict sunflower development, yield, and yield components (Chapman et al., 1993; Steer et al., 1993; Villalobos et al., 1996; Yeatts, 2004). These crop simulation models are useful tools for evaluating different agronomic management strategies (Villalobos et al., 1996). Based on a certain crop physiology background, they often adequately address the crop growth and development and their interaction with the environment. However, most of these models describe grain filling with insufficient detail, fail to take into account processes occurring inside the grain and rarely consider the accumulation of the main grain components. Some published sunflower models predict oil yield and quality but they are mainly based on empirical relationships between many traits and environmental factors and, moreover, the simulation of grain components accumulation is not dynamic (Pereyra-Irujo and Aguirrezábal, 2007; Casadebaig et al., 2011). To the best of our knowledge, a model describing the dynamics of sunflower grain filling and the accumulation of the main grain components in detail (oil and its fatty acid composition) has not been developed so far.

Non-structured models provide a trade-off between the realism of the biological processes and the relative simplicity required by modeling (Tolla et al., 2007). They are useful tools when access to the data is limited or the complexity of reactions in the pathway hinders the modeling process (Steer et al., 1993; Tolla et al., 2007; Durruty et al., 2012). The aim of the present work was to develop a non-structured mechanistic kinetic model of grain growth and oil and fatty acids biosynthesis, as a tool to describe the dynamics of grain filling in a comprehensive and quantitative way. Such a kinetic model can predict the dynamics of weight and components of the sunflower grain, and would contribute to understand the underlying mechanisms and the response of grain weight and composition to different growing conditions.

## Materials and methods

### Model development

#### General

Our model was built on the basis of biochemical reactions engineering principles. In this first version, the effect of temperature in plant development was taken into account by means of thermal time calculations. Other effects of temperature and effects of variations in daily incident radiation were not considered. Water and nutrients available are considered non-limiting for all processes. The following main assumptions were made:

- the temporal variable *t* is expressed as thermal time after flowering (accumulated degree days);
- grain is considered as a control volume that changes as the grain grows;
- grain filling is treated as a fed batch system where the grain grows in batch mode with an external carbon source;
- the external carbon source changes with time as a consequence of leaf senescence;
- once in the grain, carbon has two possible fates: (i) it turns into substrate for growth (i.e., contributes to grain weight), or (ii) it is used for maintenance.

#### Grain growth and maintenance

Logistic functions have been reportedly useful to predict the sigmoid behavior (S-shape) of sunflower grain weight dynamics (Yeatts, 2004). In the present work, a logistic equation driven by time expressed in degree days (1) was fitted to experimental data to simulate grain growth. Equation (1) was first presented by M'Kendrick and Pai (1911).

(1) $$\text{W }=\;\frac{{\text{W}}_{0}.{\text{e}}^{\mu^{\prime }.\text{t}}}{1\;-\;\frac{{\text{W}}_{0}}{{\text{W}}_{\text{max}}}\;\left(1-{\text{e}}^{\mu^{\prime }.\text{t}}\right)}$$

In this equation, *W* is the dry weight of an individual grain, W_0_ is the initial amount of W at which the model starts to work and *W*_max_ is the maximum potential grain weight. The parameter μ′ represents the specific grain growth rate (i.e., the biomass production rate per unit of biomass).

The rate at which the grain grows (*r*_*W*_) can be written as:

(2) $$\begin{array}{l} r_{W}=\mu^{\text{′}}.\frac{W_{max}\;-W}{W_{max}}.W\; \end{array}$$

The grain is not a closed system as it continuously receives the substrate assimilated by the mother plant. Once in the grain, *C* substrate rapidly reacts to form the different grain components. Thus, in a *C* mass balance, the *C* that enters the grain per unit time is equal to the consumption rate (*r*_*C*_), while the accumulation rate can be neglected. Thus *r*_*C*_ represents the rate of carbon allocation to the grains (or feed rate), here expressed as carbohydrate equivalents per degree days (Vertregt and Penning De Vries, 1987; Echarte et al., 2012).

However, not all the substrate reaching the grain is used for growth (defined as increase in grain weight). Part of this *C* is used to provide the energy needed for diverse processes that do not result in a net increase of dry weight (e.g., turnover of structures, activity of transport and movement, maintenance of concentration gradients and defense systems). The *C* costs of some of these processes are considered in calculations of carbohydrate equivalents (e.g., maintenance of the tools for biosynthesis, Vertregt and Penning De Vries, 1987). The Pirt's maintenance equation (Pirt, 1975) allows us to separate the *C* consumed by these processes from the *C* consumed for growth as follows:

(3) $$\begin{array}{l} {\text{r}}_{\text{C}}=\frac{{\text{r}}_{\text{W}}}{{\text{Y}}_{\text{G}}}\;+\text{ m}.\text{W} \end{array}$$

where Y_*G*_ and m are the actual growth yield and maintenance coefficients, respectively. The coefficient Y_*G*_ represents the biochemical efficiency of transformation of glucose into new plant material (Van Iersel and Seymour, 2000) and m, the C expended in processes that do not result in a net increase in grain dry matter. The coefficient m differs from those previously reported (Ploschuk and Hall, 1997; Van Iersel and Seymour, 2000) in that it does not consider all processes related to maintenance respiration (e.g., cell structure maintenance; Vertregt and Penning De Vries, 1987) since they were not included in carbohydrate equivalents calculations.

The first term of Equation (3) represents the substrate used for grain growth (*r*_*CG*_) and the second one the substrate used for maintenance purposes (*r*_*Cm*_). Considering total grain weight *W* as the sum of oil (W_O_) and non-oil (W_NO_) fractions, each production rate can be obtained by defining *Y*_*GO*_ and *Y*_*GNO*_ as the actual oil and non-oil fraction yield coefficients, respectively (see Supplementary Material).

#### Solar radiation interception

As the plant life progresses, the capacity of the source to feed the grain decreases because of leaf senescence, and then, *r*_*C*_ also decreases. The model takes into account this phenomenon by considering the feed rate as a function of the proportion of the photosynthetically active radiation (*p*_*PAR*_) intercepted by the crop. The value of *p*_*PAR*_ is equal to one at the initial time (beginning of grain filling) and decreases as plant leaves senesce. To solve the model, a mathematical expression of *p*_*PAR*_ is necessary. The radiation interception has been previously calculated as a function of the leaf area index, in agreement with the Lambert-Beer law, which in turn depends on thermal time (Gardner et al., 1985; Pereyra-Irujo and Aguirrezábal, 2007). *p*_*PAR*_ can be expressed as:

(4) $$\begin{array}{l} p_{PAR}=1\;-\;Kp.e^{k_{\lambda }.t} \end{array}$$

where *k*_λ_ is an empirical parameter that considers the Lambert-Beer law constant and the relationship between the leaf area index and thermal time, and *Kp* is a proportionality constant between *p*_*PAR*_ and radiation interception. In the present work, both parameters were obtained by fitting Equation (4) to experimental data.

#### Grain filling

Thus, according to the previous section and in order to take into account the contribution of *C* assimilated by the mother plant to grain filling, the theoretical accumulation of carbohydrate equivalents, i.e., the cumulative amount of substrate that enters the grain expressed by Equation (3) must be modified to:

(5) $$\begin{array}{l} \frac{\text{dC}}{\text{dt}}=\left(\frac{{\text{r}}_{\text{W}}}{{\text{Y}}_{\text{G}}}\;+\text{ m}.\text{W}\right){.\text{p}}_{\text{PAR}} \end{array}$$

On the same way, the production rate of *W* must be expressed as:

(6) $$\begin{array}{l} \frac{dW}{dt}=Y_{G}{.r}_{CG}{.p}_{PAR}=r_{W}{.p}_{PAR} \end{array}$$

Finally, according to partitioning of *C* into the oil and non-oil fractions, the production rate of each component can be depicted as:

(7) $$\begin{array}{l} \frac{dW_{O}}{dt}=Y_{GO}{.r}_{CG}{.p}_{PAR}=\frac{Y_{GO}}{Y_{G}}r_{W}{.p}_{PAR} \end{array}$$

(8) $$\begin{array}{l} \frac{dW_{NO}}{dt}=Y_{GNO}{.r}_{CG}{.p}_{PAR}=\frac{Y_{GNO}}{Y_{G}}r_{W}{.p}_{PAR} \end{array}$$

Equations (5–8) are ordinary differential equations (ODE) and must be simultaneously solved to predict the profiles of *C* and *W* during grain filling. The production rate and the substrate used for each component growth and maintenance (*r*_*WO*,_ *r*_*WNO*_, *r*_*CNO*,_ *r*_*CO*_, and *r*_*Cm*_), can also be individually predicted by coupling and solving their respective differential equations (see Supplementary Material).

#### Fatty acids biosynthesis

**Figure 1:**
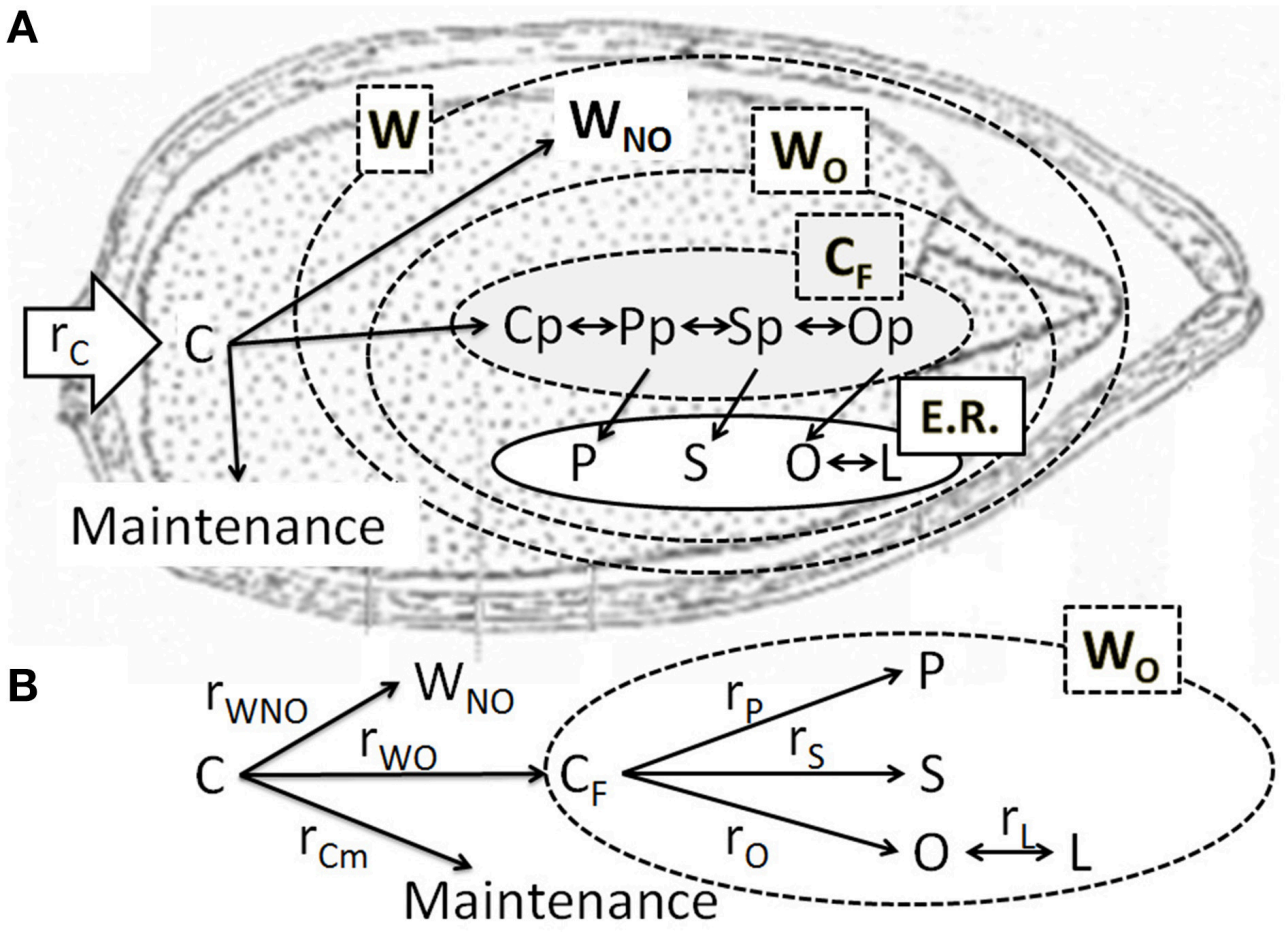
**(A)** Cartoon representing the steps proposed by the model and their localization. Once carbon (C) has been allocated to the grain it can be used for maintenance or growth. Grain weight (W) is formed by oil (W_*O*_) and non-oil (W_*NO*_) components. Compounds that react in the plastid [palmitoyl-ACP (Pp), stearoyl-ACP (Sp), and oleoyl-ACP (Op)] are grouped in a global variable C_*F*._ Fatty acids forming glycerolipidis are translocated to the endoplasmic reticulum (E.R), where the microsomal oleoylphosphatidylcholine desaturase transforms oleic acid (O) into linoleic acid (L) **(B)** Simplified reaction scheme used in this work. The parameters r_*i*_ represent the rates of production of “i” compounds. Compounds inside the dashed line form the oil fraction of grain weight.

A simplified model of fatty acids biosynthesis is shown in Figure 1. Compounds that react in the plastid are grouped in a global variable *C*_*F*_, which represents the precursor for all the fatty acids produced and stored. This is a simplification of the model presented by Martínez-Force and Garcés (2002). Lumping several intermediates into a global variable *C*_*F*_ allows considering the serial-parallel nature of the synthesis pathway without the need for a complex segregated model. Furthermore, the global variable *C*_*F*_ implicitly considers the dynamic channeling model presented by these authors.

Figure 1 shows the proposed kinetic model and the simplified reactions involved in the synthesis of fatty acids. In previous sections, substrate consumption was divided into maintenance and growth, and the grain growth was fractionated into oil and non-oil components. Since the oil fraction is composed of several fatty acids and intermediates, the rate depicted in Equation (7) pertains to the first step of fatty acids biosynthesis, i.e., the production of intermediary *C*_*F*_. The intermediate *C*_*F*_ accumulated is equal to the difference between its production from C allocated to the grain and its consumption to produce P, S, and O, and storage. In agreement with the proposed model, the following net intermediate production rate results:

(9) $$\begin{array}{l} \frac{dC_{F}}{dt}\;=\;Y_{GO}r_{CG}p_{PAR}\;-\;r_{P}\;-\;r_{O}\;-\;r_{S} \end{array}$$

Assuming the production and active transport of P, S, and O follows a specific Michaelis-Menten kinetics (i.e., they depend on grain weight, the heavier the grain, the higher the rate of synthesis of fatty acids), the production rate can be written as:

(10) $$r_{i}\;=\;\frac{v_{max}{}_{i}\left(\frac{C_{F}}{W}\right)}{Ks_{i}\;+\;\frac{C_{F}}{W}}W$$

where *v*_*max*_ and *Ks* are the maximum specific rate and half saturation constants, respectively. The subscript *i* represents P, S, or O. The fatty acid production rate is expressed here as a function of intermediate concentration (i.e., the amount of *C*_*F*_ per unit weight).

As stated above, linoleic acid is produced from oleic acid inside the endoplasmic reticulum. Then:

(11) $$r_{L}\;=\;\frac{v_{max}{}_{L}\left(\frac{O}{W}\right)}{Ks_{L}\;+\;\frac{O}{W}}W\;-\;\frac{v_{max}{}_{\;-\;L}\left(\frac{L}{W}\right)}{Ks_{\;-\;L}\;+\;\frac{L}{W}}W$$

Finally, the kinetics of *C*_*F*_ and the different fatty acids in the oleosome can be calculated via their respective mass balances.

(12) $$\frac{dC_{F}}{\text{dt}}\;=\;Y_{GO}r_{CG}p_{PAR}\;-\;\sum_{i=P,S,O}\frac{v_{max}{}_{i}\left(\frac{C_{F}}{W}\right)}{Ks_{i}\;+\;\frac{C_{F}}{W}}W$$

(13) $$\frac{\text{dP}}{dt}=\frac{v_{max}{}_{P}.\;\left(\frac{C_{F}}{W}\right)}{{\text{Ks}}_{\text{P}}\;+\frac{{\text{C}}_{\text{F}}}{\text{W}}}.\text{W}$$

(14) $$\frac{\text{dS}}{\text{dt}}=\frac{{\text{v}}_{\text{max}}{}_{\text{S}}.\;\left(\frac{{\text{C}}_{\text{F}}}{\text{W}}\right)}{{\text{Ks}}_{\text{S}}\;+\;\frac{{\text{C}}_{\text{F}}}{\text{W}}}.\text{W}$$

(15) $$\begin{array}{l} \frac{\text{dO}}{\text{dt}}=\frac{{\text{v}}_{\text{max}}{}_{\text{O}}.\left(\frac{{\text{C}}_{\text{F}}}{\text{W}}\right)}{{\text{Ks}}_{\text{O}}\text{+}\frac{{\text{C}}_{\text{F}}}{\text{W}}}.\text{W }-(\frac{{\text{v}}_{\text{max}}{}_{\text{L}}.\left(\frac{\text{O}}{\text{W}}\right)}{{\text{Ks}}_{\text{L}}\text{+}\frac{\text{O}}{\text{W}}}\text{W}\\ \;-\frac{{\text{v}}_{\text{max}}{}_{-\text{L}}.\left(\frac{\text{L}}{\text{W}}\right)}{{\text{Ks}}_{-\text{L}}\;+\;\frac{\text{L}}{\text{W}}}\text{W}) \end{array}$$

(16) $$\frac{\text{dL}}{\text{dt}}\;=\;\frac{{\text{v}}_{\text{max}}{}_{\text{L}}.\;\left(\frac{\text{O}}{\text{W}}\right)}{{\text{Ks}}_{\text{L}}\;+\;\frac{\text{O}}{\text{w}}}\text{W }-\;\frac{{\text{v}}_{\text{max}}{}_{\;-\;L}.\;\left(\frac{\text{L}}{\text{W}}\right)}{{\text{Ks}}_{\;-\;L}\;+\;\frac{\text{L}}{\text{w}}}\text{W}$$

Since *W* grows with *C*_*F*_, P, S, O, and L, the ODE Block (12–16) and differential Equation (6) must be solved simultaneously in order to predict the biosynthesis of fatty acids during grain filling.

### Experimental data

Sunflower (*Helianthus annuus* L.) was grown in the field at Balcarce Experimental Station (Unidad Integrada Balcarce INTA-FCA; 37°S, 58°W), in the Buenos Aires Province, Argentina. The model was initially calibrated with hybrid ACA885, and later evaluated with experimental data from hybrids MG2 and DK3820. The soil was a Typic Argiudoll. Experiments were performed during growing seasons 2007–2008 and 2012–2013. Each experimental unit consisted of six rows 6 m long spaced at 0.7 m. Plant population density at sowing was 6.5 plants m^−2^. The crops were grown under optimal nutrient and water conditions. Soil fertility in all experiments was adequate to attain maximum yields for sunflower crops grown under non-limiting water conditions—yield >5000 kg ha^−1^ (Sosa et al., 1999; Andrade et al., 2000). Pests, diseases and weeds were successfully controlled. Flowering of a plant was defined by the appearance of stamens in all florets from the outer whorl of the capitulum—R5.1 stage, (Schneiter and Miller, 1981).

### Sampling and chemical analysis

Sampling and chemical analysis were performed as in Echarte et al. (2013). Briefly, 12 grains of rows 6–8 were excised from the same plant as long as the total removal did not exceed 5% of the average final capitulum grain number. The number of sampling dates varied between 8 and 12, depending on the experiment. Grains were oven-dried at 60°C and weighed. Lipids were extracted with 5 ml of hexane:isopropanol (7:2, v/v) and 2.5 ml Na_2_SO_4_ (67 g l^−1^) in the presence of 0.2 ml of 1, 2, 3 triheptadecanoyl-glycerol (50 mg ml^−1^) as internal standard. The lipidic phases from the extracted samples were evaporated to dryness under a nitrogen stream. The residue was dissolved in 0.5 ml of hexane and incubated for 1 h at 80°C in the presence of the methylation mixture methanol:toluene:H_2_SO_4_ (88:10:2) and 1 ml of heptane. After samples had cooled down to room temperature, the upper phase containing fatty acids methyl esters was separated. The fatty acid composition of the extracts was determined by gas chromatography (GLC) with a Shimadzu GC-2014 chromatograph (Kyoto, Japan). The fatty acid content and total lipids extracted were calculated with the internal standard method. The oil content was assumed equal to the total extracted lipids given that they reportedly represent more than 96% of the oil (Robertson et al., 1978).

### Measurements

Global daily incident radiation was measured with pyranometers (LI-200SB, LI-COR, Lincoln, NE) from a meteorological station located ~400 m away from the experimental units. The proportion of photosynthetically active radiation (*pPAR*) intercepted by the crop at noon (±1 h) was calculated according to Gallo and Daughtry (1986) as (1 − Rb/Ro), where Rb is the radiation measured below the oldest green leaf, and Ro is the radiation measured above the canopy. Rb was measured weekly with a line quantum sensor (LI-191SB, LI-COR, Lincoln, NE, USA) positioned across the rows (the length of the sensor was modified according to the distance between rows, 0.7 m). Three measurements were taken per plot. Air temperature was measured using shielded thermistors (Cavadevices, Buenos Aires, Argentina) next to the capitulum every 60 s and averaged hourly. Measurements began after flowering and finished at physiological maturity and were recorded by data loggers (Cavadevices, Buenos Aires, Argentina).

The amount of assimilates effectively allocated to the grains (*C*) was assumed to be represented by carbon equivalents for grain biomass production (Vertregt and Penning De Vries, 1987). For this, carbon and nitrogen in the grains were determined with a TruSpec CN equipment (Leco Corporation, St. Joseph, MI), and the ash content was measured according to AOAC recommendations (Aoac, 1990). Carbohydrate equivalents for grain biomass production were calculated as described by Vertregt and Penning De Vries (1987).

### Model determinations

#### Thermal time

The temporal variable *t* is expressed as cumulative degree days, with the aim of expressing time and rates in a temperature compensated way to make temporal effects independent of temperature fluctuations (Kiniry et al., 1992; Parent and Tardieu, 2012). Cumulative degree days are calculated from daily data for mean temperature (*Tm*) and a base temperature (*Tb*) of 6°C (Kiniry et al., 1992) as follows:

(17) $$\begin{array}{l} t=\sum \left(Tm\;-\;Tb\right) \end{array}$$

#### *C_F_* intermediate

Values of *C*_*F*_ were calculated for every “*j*” thermal time from experimental data using a box mass balance Equation (18). *Y*_*WO*∕*C*_ represents the apparent oil yield coefficient, it was obtained from the linear regression of oil vs. C experimental data.

(18) $$\begin{array}{l} {C_{F}}_{t=j}=Y_{WO∕C}\;C_{t=j}\;-\;P_{t=j}\;-\;S_{t=j}{\;-\;O}_{t=j}\;-\;L_{t=j} \end{array}$$

#### Actual yield coefficients

The actual growth yield coefficient (*Y*_*G*_) and the maintenance coefficient (*m*) were calculated by linear fitting of Pirt's Equation (Pirt, 1975) to experimental data:

(19) $$\begin{array}{l} \frac{1}{Y}=\frac{1}{Y_{G}}\;+\;\frac{m}{\mu } \end{array}$$

where *Y* is the instantaneous growth yield (Pirt, 1975). Values of *Y* and μ were calculated for every “*j*” time interval as central differences of the experimental data in agreement with Equations (20) and (21), respectively.

(20) $$\begin{array}{l} Y_{t=j}=\frac{W_{t=j\;+\;1}\;-\;W_{t=j\;-1\;}}{C_{t=j\;+\;1}\;-\;C_{t=j\;-1}} \end{array}$$

(21) $$\begin{array}{l} \mu_{t=j}=\frac{dW}{dt}=\frac{W_{t=j+1}-W_{t=j-1}}{t_{j+1}-t_{j-1}} \end{array}$$

Subsequently, the actual non-oil fraction yield coefficient (*Y*_*GNO*_) and actual oil fraction yield coefficient (*Y*_*GO*_) were determined using a general non-linear regression.

#### Kinetic parameters

Growth kinetic parameters *W*_0_, *W*_*Max*_, and μ′ were calculated by non-linear fitting of Equation (1). The kinetic parameters for fatty acids production *v*_*max*_ and *Ks* were obtained by non-linear fitting of Equations (10) and (11). The values of production rates were obtained by finite differences (central differences) according to Equation (22):

(22) $$\begin{array}{l} {r_{i}}_{t=j}\;=\;\frac{di}{dt}\;=\;\frac{i_{t=j\;+\;1}\;-\;i_{t=j-1}}{t_{j\;+\;1}\;-\;t_{j-1}} \end{array}$$

where *i* represent P, S, O, or L. Once all the parameters were obtained, a further step of model optimization was performed using a general non-linear regression framework.

### Sensitivity analysis

A sensitivity analysis was performed as in Villalobos et al. (1996). The effects of ±25% variation in every kinetic parameter of the model on the main variables output (C, W, P, S, O, and L) were analyzed. The sensitive coefficient (*SC*) was calculated as in Equation (23), being *V* the output variable and *P* the kinetic parameter.

(23) $$\begin{array}{l} SC=\frac{\Delta V∕V}{\Delta P∕P} \end{array}$$

### Informatics tools and statistics

Linear and non-linear regressions were performed with Origin 8.0® (OriginPro, v. 8.0724; OriginLabCorporation, Northampton, MA 01060, USA). Once the kinetic parameters were obtained, profiles were modeled using a fourth-order Runge-Kutta algorithm coupled to the regression in order to integrate the differential equations simultaneously (MathCad 14.0.0.163, Parametric Technology Corporation). The goodness-of-fit of the model was evaluated using the regression coefficient (*R*^2^) and Reduced Chi-square (χ^2^) test via the Prob (χ^2^ > *F*) with α < 0.05. Significant differences among parameters were evaluated by Student *t*-test (95% confidence interval).

## Results

### Solar radiation interception

**Figure 2:**
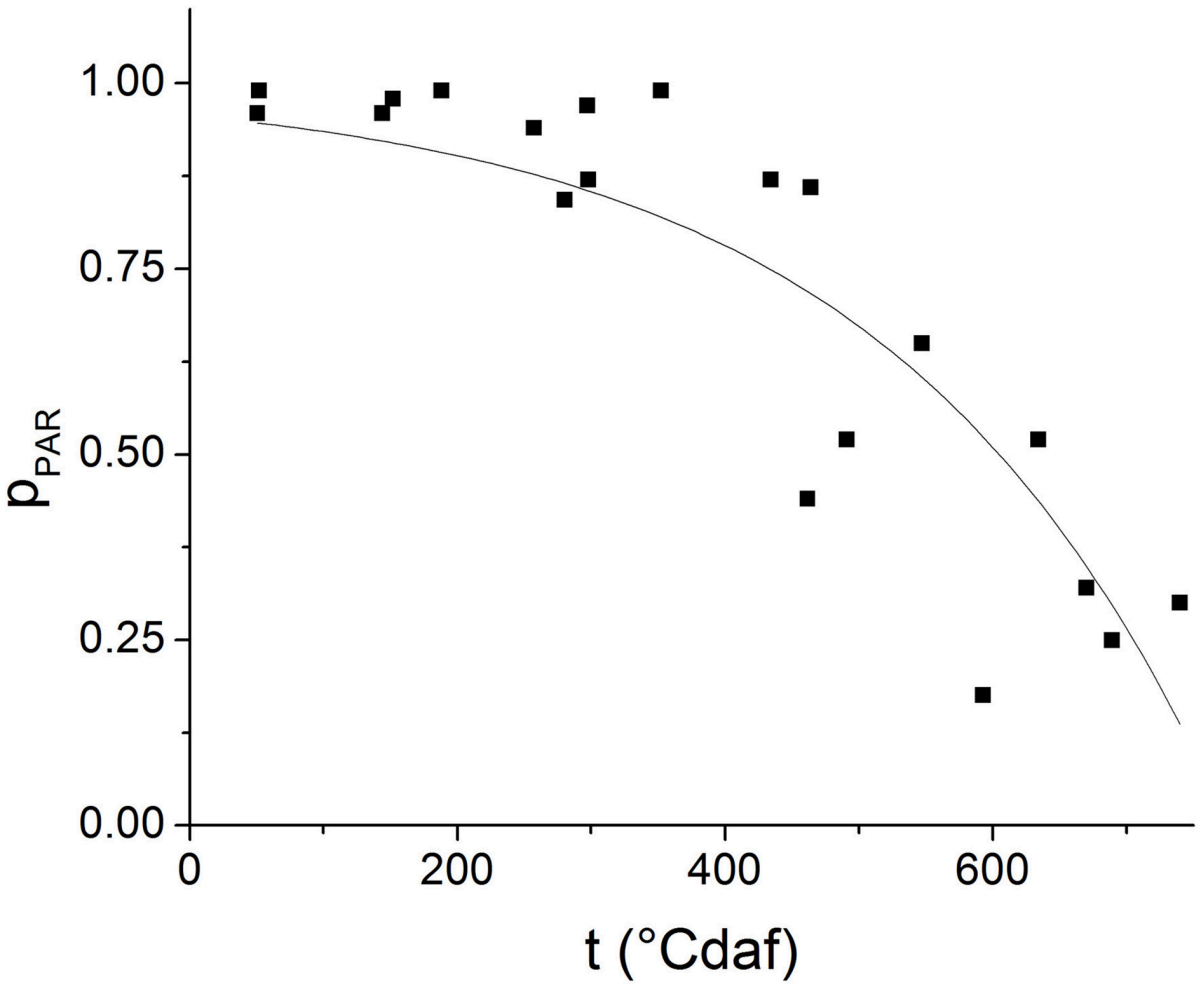
**Proportion of photosynthetically active radiation intercepted by the plants (***p***_***PAR***_) along grain filling**. The symbols represent observed data and the lines represent the results of fitting Equation (4) to experimental data.

Figure 2 shows the experimental values of *p*_*PAR*_ as a function of degree days after flowering for several experiments. The line represents the result of fitting Equation (4) to the experimental data (*R*^2^ = 0.7633, *P* < 0.001). The values obtained for the empirical parameter *k*_λ_ and the proportionality constant *Kp* resulted 4.04 × 10^−3^ ± 7.12 × 10^−4^ °Cd af^−1^ and 0.0434 ± 0.0199, respectively.

### Grain growth and filling

**Figure 3:**
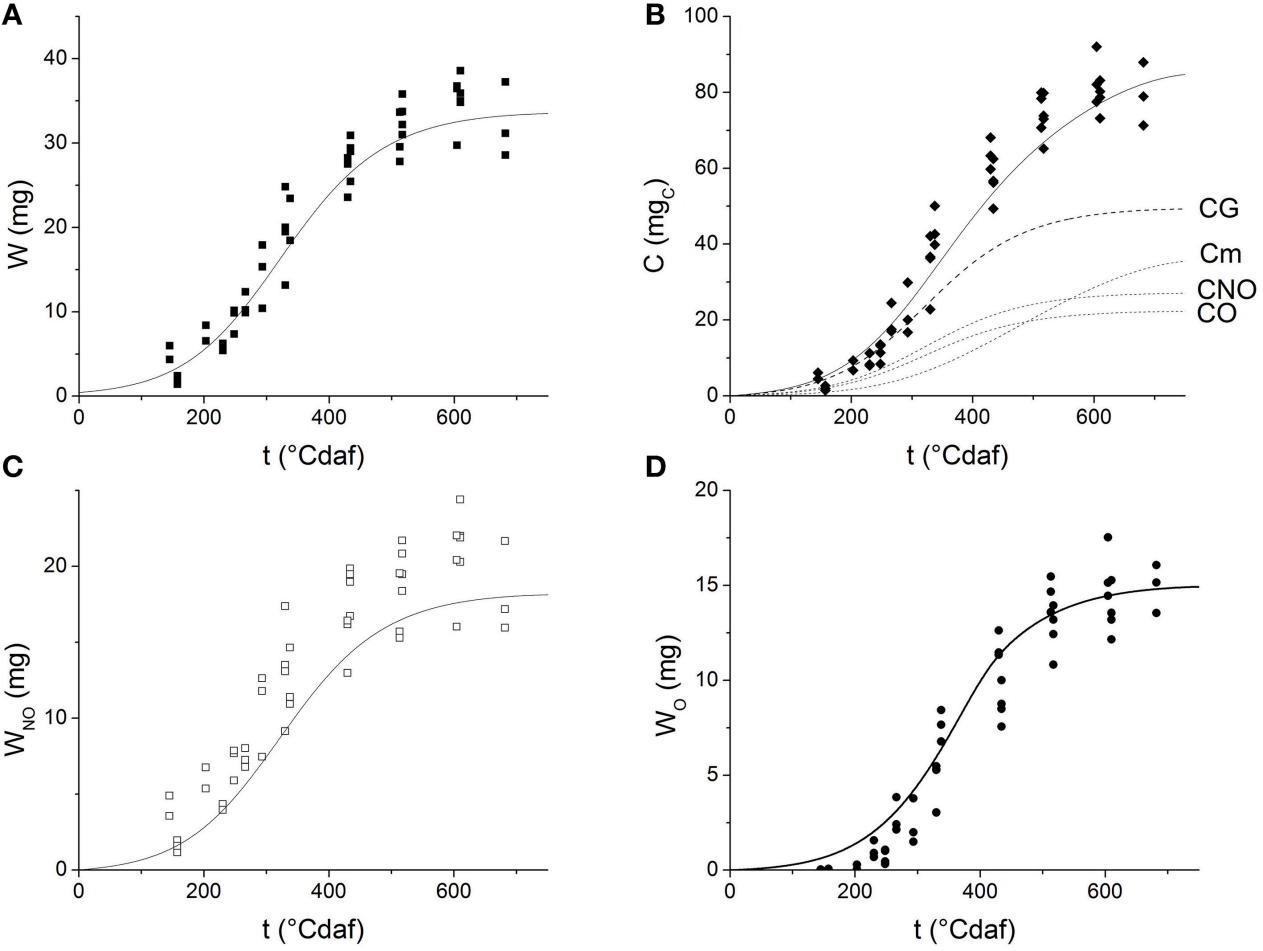
**Dynamics of grain weight and its components as a function of thermal time after flowering: (A) grain weight; (B) cumulative carbohydrate equivalents; (C) non-oil weight (D) oil weight**. Symbols represent experimental data. Solid lines represent the values simulated by the grain filling model. The dashed lines on Figure 3B represent carbohydrate equivalents consumed for maintenance purposes (Cm), to produce grain (CG), non-oil fraction (CNO), and oil fraction (CO).

Grains grow with thermal time following a sigmoid curve (Figure 3A). After fitting Equation (1) to the experimental data, the values obtained for *W*_0_, *W*_*max*_ and μ′ were 0.4299 ± 0.1634 mg, 34.5396 ± 0.9131 mg, and 0.0145 ± 0.0013 °Cdaf^−1^, respectively [*R*^2^ = 0.94547, Prob (χ^2^ > *F*) = 0]. Figure 3B shows the experimental values of C vs. thermal time. The values obtained for *Y*_*G*_ and *m* by fitting Equation (19) to the experimental data were 0.671 ± 0.036 mg ${\text{mg}}_{\text{C}}^{-1}$ and 4.301 × 10^−3^ ± 4.28 × 10^−4^ mg_C_ mg^−1°^Cdaf^−1^, respectively. The fitted values for *Y*_*GNO*_ and *Y*_*GO*_ were 0.368 ± 0.021 g ${\text{g}}_{\text{C}}^{-1}$ and 0.303 ± 0.016 g ${\text{g}}_{\text{C}}^{-1}$, respectively [*R*^2^ = 0.9552, Prob(χ^2^ > *F*) = 0].

Grain weight and cumulative carbohydrate equivalents were simulated by solving simultaneously ODE Block (5–8) with the previously fitted parameters. The values predicted by the model are presented in Figures 3A,B as solid lines. The figures indicate the model successfully describes the experimental grain growth kinetics and it is able to predict the theoretical cumulative amount of carbohydrates that is allocated to the grain during the filling period. The amount of substrate spent in growth (CG), maintenance (Cm), oil (CO), and non-oil (CNO) grain fractions were also predicted and plotted as a function of thermal time (dashed lines in Figure 3B). Different dynamics of carbon investment were observed: at first, most of the substrate is used to produce grain mass, later, the maintenance requirements increase and thus less substrate is used for growth.

Values of W_NO_ and W_O_ were also obtained by solving ODE Block (5–8) (Figures 3C,D). The simulations seem to overestimate the experimental *W*_*O*_-values before 200°Cdaf.

**Figure 4:**
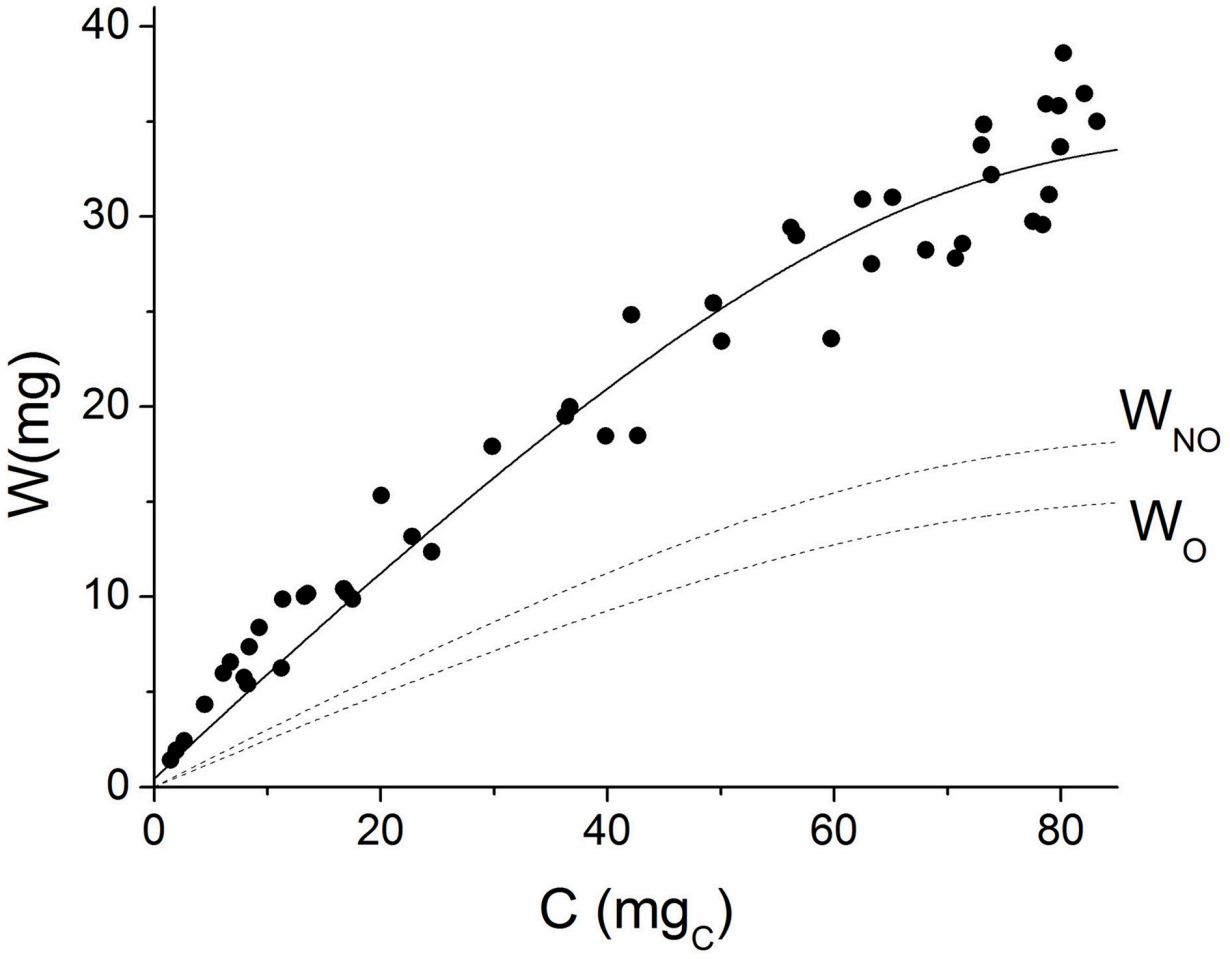
**Grain weight vs. cumulative carbohydrate equivalents**. Symbols represent experimental data. Solid line represents the values predicted by the model. Dashed lines represent the values predicted for oil (W_O_) and non-oil grain fraction (W_NO_).

Grain weight was plotted as a function of carbohydrate equivalents in Figure 4. The solid line shows the values predicted by the model; the instantaneous growth yield is represented by the slope of this line. It can be observed that the model acceptably simulates the non-linear behavior of experimental data. At the beginning of grain filling, when grains are small, they present lower maintenance requirements, which results in a higher apparent growth yield. As grains grow in size, more substrate is used for maintenance and the apparent yield coefficient decreases.

### Fatty acids biosynthesis

**Figure 5:**
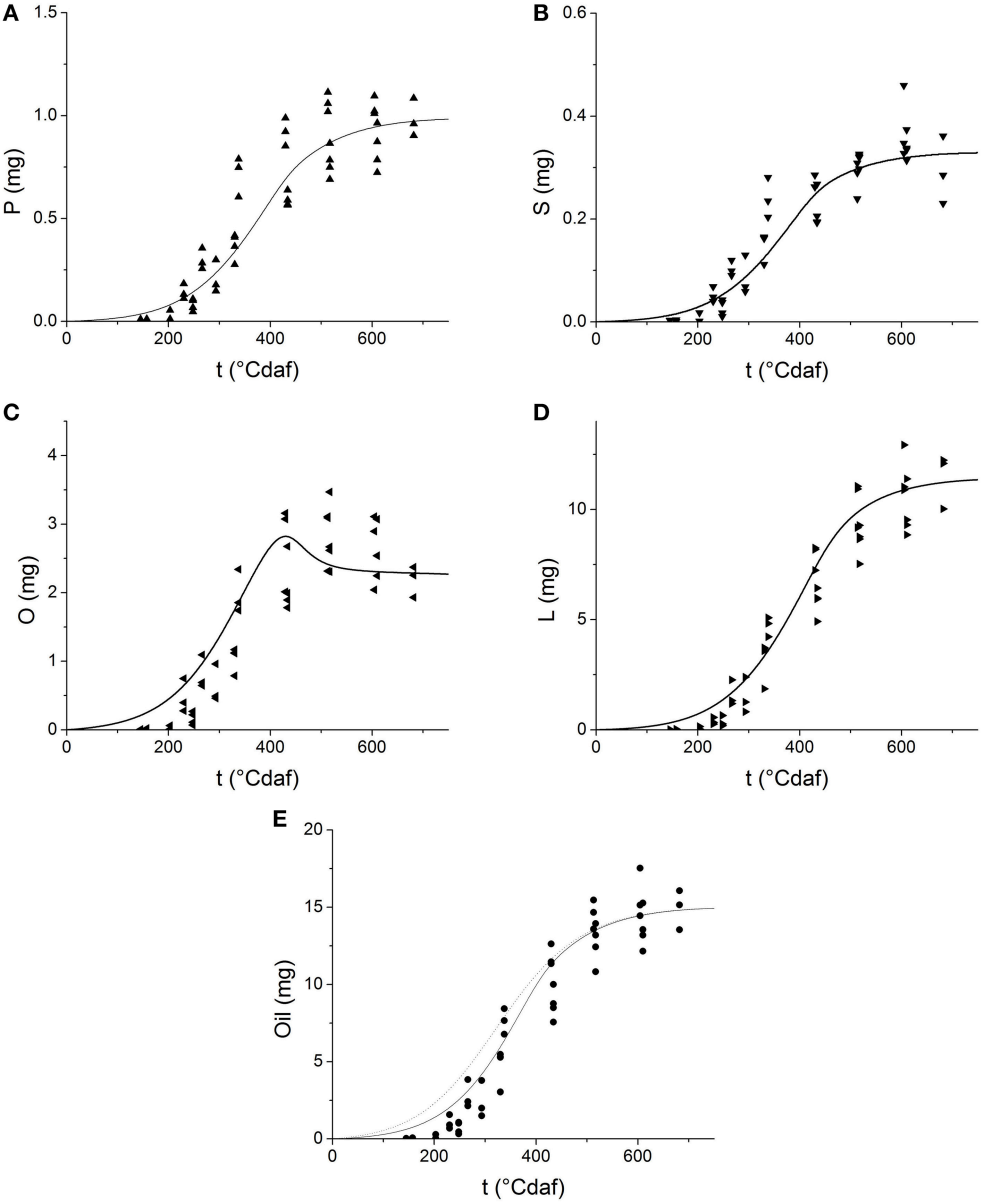
**Dynamics of fatty acid as a function of thermal time after flowering: (A) palmitic acid; (B) stearic acid; (C) oleic acid; (D) linoleic acid; (E) total fatty acids**. Symbols represent experimental data. Solid lines represent the values predicted by the grain filling model. The dotted line on Panel **(E)** represents the simulation of oil grain weight by a single step first approach.

Table 1 shows the kinetic parameters obtained from fitting Equation (10) and (11). The values predicted by the model are presented in Figure 5 as solid lines. The model successfully describes the experimental behavior of all fatty acids. The rates of production of every fatty acid increase early during grain filling together with grain weight, later they decrease until they completely stop. The model predicts the amount of oleic acid increases at the early stages of grain filling, reaches a maximum at 400°Cdaf and then decreases. Linoleic acid production follows an end-product saturating specific kinetics, like palmitic, and stearic acid, but smoothed and delayed by a reversible reaction and by the fact that linoleic acid is the final product of three serial steps (C→C_F_ →O→L).

**Table 1:** **Kinetic parameters of fatty acids biosynthesis for ACA885 sunflower hybrid**.

**Product**	**Parameter**	**Value ± *SD***	**Prob(χ^2^ < *F*)**
Palmitic acid	v_max_	2.025 × 10^−4^± 6.769 × 10^−5^	4.71E-4
	Ks	0.0139 ± 0.022	
Stearic acid	v_max_	7.783 × 10^−5^ ± 2.814 × 10^−5^	2.04E-4
	Ks	0.0214 ± 0.0029	
Oleic acid	v_max_	3.157 × 10^−3^ ± 8.463 × 10^−4^	2.76E-5
	Ks	0.0199 ± 0.0025	
Linoleic acid	v_max_	0.0197 ± 2.646 × 10^−3^	3.15E-8
	Ks	0.0202 ± 0.0322	
	v_max−1_	0.0190 ± 6.837 × 10^−4^	
	Ks_−1_	0.0159 ± 0.00221	

Once the profile of each fatty acid was obtained, the concentration of oil was calculated as the sum of all fatty acids and plotted in Figure 5E as a solid line. The dotted line in the figure represents the oil grain weight predicted in a first approach (Figure 3D), where a single step production process was considered (see Section Grain Growth and Filling). A better performance of the model was achieved when the serial nature of fatty acids biosynthesis was considered, especially at short times (<300°Cdaf) when *C*_*F*_ is accumulated.

### Sensitivity analysis

A sensitivity analysis was performed on the proposed model. The effect of ±25% variation in every kinetic input parameter on the model output was observed at three different times during grain filling. The results, presented in Table 2, show that the relative influence of the parameters on both grain weight and composition varies along the grain development. Specific growth (μ′) has significant influence on all the variables at the beginning of grain filling (*SC* > 0.5) but its effect decays later on (*SC* < 0.5), when C is used in maintenance processes. On the other hand, W_0_, W_max_, Y_G_, and *m* exert low influence (*SC* < 0.3) on most of the variables. However, palmitic, stearic, and linoleic acids were sensitive to grain growth and oil yield (*Y*_*G*_ and *Y*_*GO*_), showing the influence of the partitioning of carbon to the oil or non-oil fraction on fatty acid composition. Saturated fatty acids were sensitive to their own maximum specific rate of synthesis (*v*_*maxS*_ and *v*_*maxP*_) and to the oleic acid maximum specific rate (*v*_*maxO*_), while oleic acid was mostly influenced by parameters driving the synthesis of linoleic acid.

**Table 2:** **Sensitivity coefficients of parameters for ACA885 sunflower hybrid dynamics**.

		**Positive change**						**Negative change**					
**Parameter**	**T**	**C**	**W**	**P**	**S**	**O**	**L**	**C**	**W**	**P**	**S**	**O**	**L**
**SENSITIVITY COEFFICIENT (SC)**													
μ′	300	0.54	0.522	0.564	0.58	0.57	0.569	0.594	0.593	0.549	0.557	0.579	0.54
	600	0.125	0.046	0.034	0.055	0.008	0.059	0.344	0.27	0.274	0.285	0.148	0.311
	800	0.103	0.023	0.004	0.029	0.024	0.025	0.282	0.184	0.162	0.193	0.185	0.188
W_0_	300	0.125	0.119	0.158	0.159	0.144	0.164	0.203	0.197	0.237	0.228	0.224	0.245
	600	0.03	0.011	0.01	0.008	–	0.01	0.055	0.024	0.024	0.018	0.001	0.025
	800	0.024	0.005	0.003	0.003	0.005	0.002	0.044	0.012	0.008	0.011	0.011	0.008
W_max_	300	0.07	0.075	0.041	0.047	0.055	0.039	0.129	0.138	0.084	0.089	0.104	0.076
	600	0.179	0.201	0.201	0.205	0.217	0.2	0.256	0.28	0.281	0.285	0.294	0.281
	800	0.186	0.209	0.211	0.211	0.21	0.212	0.263	0.287	0.291	0.293	0.288	0.292
Y_G_	300	−0.138	–	−0.045	−0.056	−0.071	−0.054	−0.312	–	−0.042	−0.063	−0.068	−0.051
	600	−0.109	–	−0.152	−0.180	−0.069	−0.198	−0.245	–	−0.338	−0.407	−0.145	−0.449
	800	−0.101	–	−0.153	−0.180	−0.055	−0.199	−0.228	–	−0.339	−0.408	−0.080	−0.458
m	300	0.044	–	–	–	–	–	0.06	–	–	–	–	–
	600	0.081	–	–	–	–	–	0.11	–	–	–	–	–
	800	0.091	–	–	–	–	–	0.123	–	–	–	–	–
Y_GO_	300	–	–	0.024	0.037	0.042	0.031	–	–	0.084	0.114	0.134	0.102
	600	–	–	0.188	0.227	0.076	0.25	–	–	0.258	0.303	0.121	0.334
	800	–	–	0.188	0.225	0.047	0.253	–	–	0.26	0.304	0.098	0.336
v_maxP_	300	–	–	0.217	–	−0.002	−0.001	–	–	0.293	−0.013	−0.003	−0.002
	600	–	–	0.202	−0.014	−0.005	−0.016	–	–	0.281	−0.022	−0.008	−0.022
	800	–	–	0.202	−0.016	−0.004	−0.016	–	–	0.281	−0.018	−0.005	−0.023
Ks_P_	300	–	–	−0.017	–	−0.012	0.007	–	–	−0.023	–	−0.017	0.01
	600	–	–	−0.052	0.003	−0.007	0.006	–	–	−0.091	0.007	−0.009	0.01
	800	–	–	−0.057	0.003	−0.008	0.006	–	–	−0.101	0.007	−0.010	0.011
v_maxS_	300	–	–	–	0.224	−0.001	–	–	–	–	0.291	−0.002	−0.001
	600	–	–	−0.004	0.211	−0.002	−0.005	–	–	−0.006	0.289	−0.003	−0.007
	800	–	–	−0.004	0.211	−0.002	−0.006	–	–	−0.006	0.29	−0.002	−0.007
Ks_S_	300	–	–	–	−0.019	−0.019	0.011	–	–	–	−0.038	−0.027	0.015
	600	–	–	0.002	−0.060	−0.013	0.004	–	–	0.003	−0.107	−0.018	0.007
	800	–	–	0.001	−0.063	−0.013	0.004	–	–	0.002	−0.114	−0.018	0.007
v_maxO_	300	–	–	−0.027	−0.037	0.194	0.162	–	–	−0.023	−0.038	0.274	0.27
	600	–	–	−0.142	−0.167	0.004	0.017	–	–	−0.306	−0.359	0.005	0.038
	800	–	–	−0.143	−0.167	0.003	0.016	–	–	−0.309	−0.358	0.008	0.035
Ks_O_	300	–	–	0.003	0.009	−0.039	−0.009	–	–	0.005	–	−0.058	−0.013
	600	–	–	0.055	0.057	−0.015	−0.004	–	–	0.089	0.089	−0.021	−0.007
	800	–	–	0.061	0.061	−0.014	−0.004	–	–	0.097	0.097	−0.019	−0.007
v_maxL_	300	–	–	–	–	−0.45	0.249	–	–	–	–	–1.235	0.682
	600	–	–	–	–	−0.453	0.096	–	–	–	–	–3.642	0.775
	800	–	–	–	–	−0.443	0.088	–	–	–	–	–3.859	0.764
Ks_L_	300	–	–	–	–	0.13	−0.072	–	–	–	–	0.192	−0.107
	600	–	–	–	–	0.214	−0.046	–	–	–	–	0.277	−0.059
	800	–	–	–	–	0.215	−0.043	–	–	–	–	0.278	−0.055
v_max−L_	300	–	–	–	–	0.701	−0.387	–	–	–	–	0.642	−0.355
	600	–	–	–	–	2.003	−0.426	–	–	–	–	0.694	−0.148
	800	–	–	–	–	2.056	−0.407	–	–	–	–	0.687	−0.136
Ks_−L_	300	–	–	–	–	−0.063	0.035	–	–	–	–	−0.103	0.057
	600	–	–	–	–	−0.047	0.01	–	–	–	–	−0.073	0.016
	800	–	–	–	–	−0.045	0.009	–	–	–	–	−0.068	0.014

*Positive and negative change refers to the fitted kinetic parameter ±25% variation, respectively*.

### Model extrapolation to different hybrids

The ability of the model to predict the behavior of two independent hybrids that were not used for model calibration (MG2 and DK3820) was evaluated. Parameter values and their comparison among hybrids are provided as Supplementary Material (Table SM1). In order to define the smallest set of values necessary to obtain an appropriate simulation, the model was run by: (i) freely fitting all the parameters, or (ii) fitting the minimum amount of parameters that would ensure a good predictive quality [Prob(χ2 > *F*) < 0.01]. For this, parameters of low sensitivity (W_0_, W_max_, *Y*_*G*_, *m*, Ks_i_) and μ′ (sensitive only at the beginning of grain filling and low genetic variability) were fixed and *v*_*maxi*_ parameters were refitted. In (ii), the ACA885 parameter values were used instead of each hybrid's own parameters (bold values in Table 3). Table 3 summarizes the kinetic parameters obtained by fitting the model to the experimental values measured for all hybrids.

**Table 3:** **Kinetic parameters for different hybrids (MG2 and DK3820)**.

**Parameter**	**MG2 (i)**	**MG2 (ii)**	**DK3820 (i)**	**DK3820 (ii)**
*μ′* [°Cdaf^−1^]	0.0126 ± 0.0017	**0.0145 ± 0.0013**	0.018 ± 0.003	**0.0145 ± 0.0013**
W_0_ [mg]	0.5862 ± 0.1906	**0.4299± 0.1634**	0.2400 ± 0.2273	**0.4299± 0.1634**
W_max_ [mg]	37.5079 ± 1.2389	**34.5396 ± 0.9131**	39.35186 ± 1.67339	**34.5396 ± 0.9131**
Y_G_ [mg.mg${}_{\text{C}}^{-1}$]	0.950 ± 0.082	**0.671 ± 0.036**	0.638 ± 0.145	**0.671 ± 0.036**
m [mg_C_.mg^−1^.°Cdaf^−1^]	4.25 × 10^−3^ ± 7.69 × 10^−4^	**4.31** × **10^−3^ ± 4.28** × **10^−4^**	2.43 × 10^−3^ ± 1.37 × 10^−4^	**4.31** × **10^−3^ ± 4.28** × **10^−4^**
Y_GO_ [mg.mg${}_{\text{C}}^{-1}$]	0.406 ± 0.035	**0.303 ± 0.016**	0.208 ± 0.048	**0.303 ± 0.016**
v_maxP_ [mg.°Cdaf-1]	1.455 × 10^−4^ ± 6.815 × 10^−5^	2.531 × 10^−4^ ± 9.856 × 10^−5^	3.581 × 10^−4^ ± 9.157 × 10^−5^	1.758 × 10^−4^ ± 7.492 × 10^−5^
Ks_P_ [mg.mgW-1]	0.0106 ± 0.00137	**0.0139 ± 0.0022**	0.0755 ± 0.01855	**0.0139 ± 0.0022**
v_maxS_ [mg.°Cdaf-1]	1.6004 × 10^−4^ ± 1.107 × 10^−5^	2.058 × 10^−4^ ± 1.925 × 10^−5^	3.315 × 10^−4^ ± 9.216 × 10^−5^	1.414 × 10^−4^ ± 7.890 × 10^−5^
Ks_S_ [mg.mgW-1]	0.04537 ± 0.00114	**0.0214 ± 0.0029**	0.1336 ± 0.04891	**0.0214 ± 0.0029**
v_maxO_ [mg.°Cdaf-1]	3.410 × 10^−3^ ± 1.683 × 10^−4^	4.095 × 10^−3^ ± 2.021 × 10^−4^	3.912 × 10^−3^ ± 1.345 × 10^−4^	4.116 × 10^−3^ ± 1.452 × 10^−4^
Ks_O_ [mg.mgW-1]	0.0415 ± 0.0012	**0.0199 ± 0.0025**	0.03778 ± 0.00301	**0.0199 ± 0.0025**
v_maxL_ [mg.°Cdaf-1]	0.0367 ± 5.049 × 10^−3^	0.0405 ± 5.571 × 10^−3^	0.0440 ± 7.093 × 10^−3^	0.0357 ± 8.742 × 10^−3^
Ks_L_ [mg.mgW-1]	0.0595 ± 0.0017	**0.0202 ± 0.0322**	0.0423 ± 0.0052	**0.0202 ± 0.0322**
v_max−L_ [mg.°Cdaf-1]	0.0291 ± 4.032 × 10^−4^	0.0381 ± 5.241 × 10^−4^	0.0349 ± 6.121 × 10^−4^	0.0295 ± 5.221 × 10^−4^
Ks_−L_ [mg.mgW-1]	0.0391 ± 0.0021	**0.0159 ± 0.0022**	0.0723 ± 0.0036	**0.0159 ± 0.0022**

*The model was run by: (i) freely fitting all the parameters or (ii) fitting the minimum amount of parameters that would ensure a good predictive quality (Prob(χ2 > F) < 0.01). Highlighted (bold) values represent parameters obtained for ACA885*.

**Figure 6:**
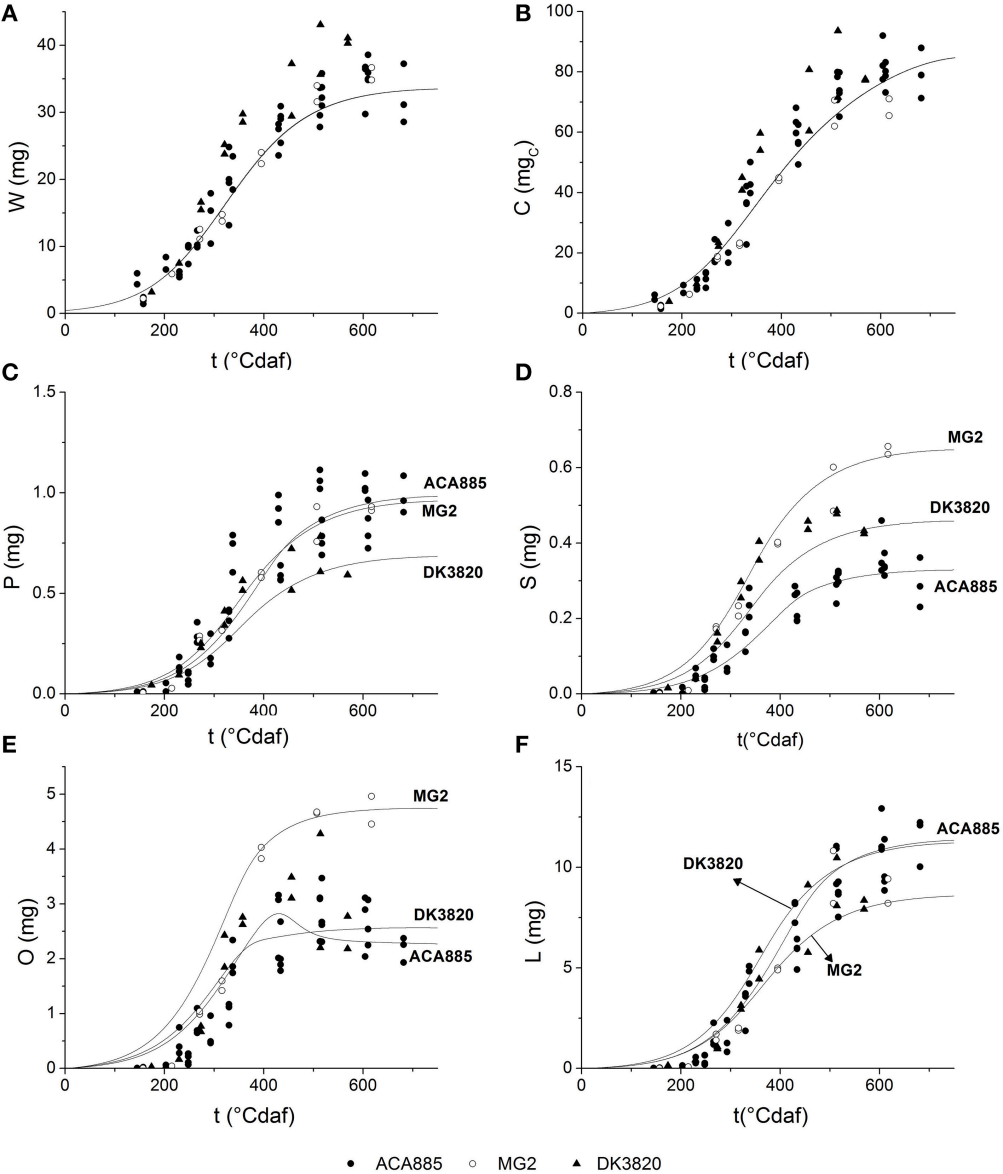
**Grain filling dynamics of different hybrids**. Simulations were done using parameters set (ii) depicted in Table 3: **(A)** grain growth **(B)** carbohydrate equivalents; **(C)** palmitic acid; **(D)** stearic acid; **(E)** oleic acid; **(F)** linoleic acid. ACA885, closed circles; MG2, open circles; DK3820, triangles. Solid lines represent the model predictions.

Growth parameter values are similar for all hybrids, with the exception of *Y*_*G*_ and *Y*_*GO*_ of MG2. Parameter μ′ showed high sensitivity early during grain filling but did not differ among hybrids, while *v*_*maxO*_ showed both low sensitivity and low genetic variability. When the set of parameters (i) was used, the model successfully predicted the kinetics of every trait explored in this research (data not shown). Grain weight, theoretical accumulated carbohydrates, and fatty acid composition dynamics simulated by fitting the minimal amount of parameters (case ii in Table 3) are shown in Figure 6. In this case, simulated values of C and W adequately described the experimental behavior of both MG2 and DK3820 hybrids (Figures 6A,B). On the other hand, the extrapolation of all fatty acids biosynthesis parameters was not possible due to the high sensitivity of P, S, O, and L to *v*_*maxi*_. The model adequately predicted fatty acids dynamics during grain filling when five out of the 16 parameters were refitted.

## Discussion

In the present work, a non-structured mechanistic kinetic model of grain growth and oil and fatty acids biosynthesis has been developed. By setting initial conditions –*W*_0_ for grain weight– and calculating carbon assimilated by leaves and allocated to the grains as the substrate, the oil, and non-oil weight and oil composition dynamics have been successfully simulated for different sunflower hybrids. To the best of our knowledge, a model with the ability of describing the grain filling dynamics in such detail has not been previously developed for sunflower, or any other crop species.

### Carbon partitioning to growth and maintenance

The model considered that the carbon substrate was destined to both growth and maintenance. The amount of substrate that was actually transformed into grain biomass (*Y*_*G*_) was in the range of previously reported values for conversion efficiency (0.6 to 0.8 mg mg^−1^ Mccree, 1982; Van Iersel and Seymour, 2000). The maintenance coefficient (*m*) was as well in the range of reported values [0.003 to 0.050 mg mg^−1^ d^−1^ (Hesketh et al., 1980)], despite in the present work it did not consider the carbon costs of cell structure maintenance (Penning De Vries et al., 1974). High variability of *m* has been associated to the dependence of this coefficient on the age of the plant and the environmental conditions during grain filling (Van Iersel and Seymour, 2000).

The results show that if maintenance processes were negligible, 45% of the carbohydrates destined for growth would be transformed into oil. When maintenance processes were considered, 45% of the carbohydrates allocated were destined to grain growth and only 40% of them were converted into oil. Therefore, the results of the model indicate that maintenance processes not only reduce the grain growth, but also the selectivity to oil. In light of these findings, further research might help to understand the physiological processes underlying the relationship between maintenance and grain composition.

**Figure 7:**
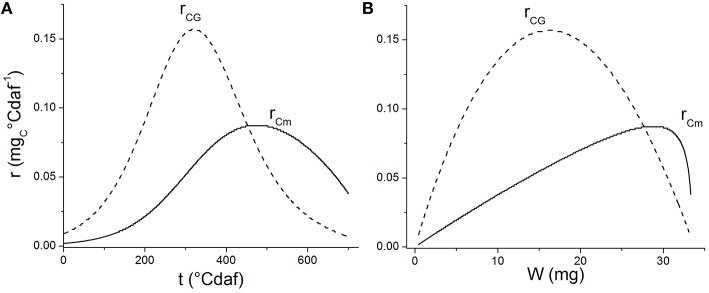
**Rates of substrate consumption for maintenance (r_***Cm*****,**_ solid lines) and substrate consumption for growth (r_***CG***_, dashed lines) vs. thermal time (A) and grain weight (B)**.

Carbon partitioning to maintenance or growth changed with ontogeny. As the grain grew, the substrate destined to maintenance increased (Figure 3C) and the apparent yield coefficient decreased, in agreement with Pirt's law, (Equation 3; Pirt, 1975). Van Iersel and Seymour (2000) found that *r*_*Cm*_ depends on both the age of the plant and the biomass dry weight. A similar behavior was found when analyzing the consumption rate of substrate for maintenance (*r*_*Cm*_) or grain growth (*r*_*CG*_) as a function of thermal time (Figure 7A) or grain weight (Figure 7B; see Supplementary Material for *r*_*Cm*_ and *r*_*CG*_ calculation). The value of *r*_*CG*_ presents a maximum at 300°Cdaf, in concordance with the inflection point of the sigmoid growth curve (Figure 3A). The value of *r*_*Cm*_ reaches its maximum later and at higher grain weight than *r*_*CG*_ (Figure 7B). The earlier decrease of *r*_*CG*_ indicates the system is more selective to maintenance when the grain is bigger. In this sense, Van Iersel and Seymour (2000) propose that younger plants do not show substrate limitations and maintenance increases as the grain grows. The amount of substrate consumed for growth (*r*_*CG*_) increases with *W* due to higher carbon use efficiency as the plants become bigger. As the plant life progresses (later in the plant cycle and higher *W*), the substrate available diminishes together with the substrate destined for both, growth and maintenance. According with Figure 1A when *r*_*C*_ falls due to *p*_*par*_ effect, both *r*_*Cm*_ and *r*_*CG*_ fall because they are also limited by carbon allocation (Equation 5).

#### Simulation of grain weight and composition dynamics

Grain growth (total weight, oil, and non-oil components) followed sigmoid functions with time, in agreement with many reports in the literature (Aguirrezábal et al., 2003; Mantese et al., 2006; Rondanini et al., 2007; Echarte et al., 2013). In a previous work, Echarte et al. (2012) reported that the grain weight and oil content linearly increased with the amount of carbon allocated to the grains. In this research, a model with a more mechanistic approach was able to predict the theoretical cumulative amount of carbohydrates that was allocated to the grain during the filling period, and successfully described the grain growth kinetics.

The kinetic parameters of fatty acid biosynthesis were first obtained for sunflower hybrid ACA885. Given a parallel reaction scheme, the system is considered more selective toward the reaction with higher rate. A higher maximum rate of production for oleic acid (*v*_*maxO*_) than for palmitic and stearic acids made the system more selective toward oleic acid. Similar values of Ks for P, S, and O, which represent the affinity of enzymes involved in their active transport out of the plastid, suggest that these enzymes have similar affinity for the three fatty acids. According to the model predictions, palmitic, and stearic acids followed typical end-product saturating specific kinetics (Figures 5A,B), while oleic acid increased at early stages of grain filling up to a maximum. These predictions are in agreement with previous studies (Martínez-Force et al., 1998; Santonoceto et al., 2003; Echarte et al., 2013). One possible explanation for the behavior of oleic acid is that as grain filling progresses, oleate desaturase activity increases (Gray and Kekwick, 1996), but carbon accumulates in the grain faster than the increment of this catalytic activity. Therefore, between 150 and 300°Cdaf, oleic acid begins to accumulate. Between 300 and 350°Cdaf, a high desaturation activity produces a decrease of oleic acid with a concomitant increase of linoleic acid, being more evident when carbon is scarce (Echarte et al., 2013). The accumulation of linoleic acid responds to higher values of *v*_*maxL*_ than _*Vmax*−*L*_. Furthermore, *v*_*maxL*_ was the highest *v*_*max*_ value of all, explaining the high productivity of this compound in agreement with previously reported data (Martínez-Force et al., 1998; Santonoceto et al., 2003; Echarte et al., 2013).

In a first approach, the oil fraction was predicted as if it were produced in a single reaction step (Figure 3D), although it is the final product of a more complex reaction pathway. The low oil fraction values estimated this way suggest an accumulation of intermediate compounds, that were assigned to the non-oil fraction in this first approach. However, when total fatty acids (oil) were estimated as the sum of every fatty acid predicted (Figure 5E) a better performance was achieved. Thus, the model depicted in Figure 1, which considers the serial nature of fatty acids biosynthesis, was able to better represent the experimental behavior, especially at short times (<300°Cdaf) when *C*_*F*_ is accumulated.

#### Finding key genotype parameters of the model

Complex models are not suitable for the characterization of the dynamics of multiple genotypes, since once the model has been built, finding the kinetic parameters of a new hybrid might be laborious, expensive, and time consuming. Thus, many models rely on a limited number of genetic parameters that appropriately describe one particular genotype behavior, while assuming that the rest of the parameters do not significantly influence the model output for any genotype (Quilot et al., 2005; Makowski et al., 2006). Given a small set of parameters, re-parameterizing many growing models may not require new dynamic measurements, and parameters can be estimated from the final values by optimization methods. The model developed here for sunflower hybrid ACA855 used 16 input parameters to simulate grain weight and component dynamics. Combining the results of sensitivity analysis and the genetic variability of parameters of two other hybrids (MG2 and DK3820), the number of model input parameters was reduced to five. Whether this is sufficient to simulate the behavior of the universe of commercial hybrids should be further tested working with a bigger pool of hybrids than the one explored in this research.

A sensitivity analysis showed that the influence of parameters changed with ontogeny. Although the specific growth rate (μ′) was the most influential grain growth parameter on model output at the beginning of grain filling, similar values among hybrids indicate low genetic variability and thus, a unique value of μ′ could satisfactorily simulate grain filling dynamics for any hybrid. Although they are parameters of low sensitivity, higher values of conversion efficiencies (*Y*_*G*_ and *Y*_*GO*_) for MG2 suggest this hybrid devotes more substrate to grain weight than the other hybrids.

Fitting of parameters related to fatty acids biosynthesis, more specifically the maximum specific rates (*v*_*maxP*_, *v*_*maxS*_, *v*_*maxO*,_ *v*_*maxL*_, *v*_*max*−*L*_), is needed to re-calibrate the model for every hybrid to adequately predict the fatty acid composition. Traditional commercial hybrids have been improved to obtain maximal grain weight and oil content, but not targeting their fatty acid composition. The latter could have been modified or unintentionally selected when domesticating or breeding other characters (Chapman and Burke, 2012). Hybrids with different potential fatty acid composition have been obtained by mutagenesis (e.g., high oleic hybrids) and oils with certain fatty acid composition receive a prime over the regular price in the market. This model could help to understand the dynamics of oil and fatty acid biosynthesis in sunflower hybrids with modified potential fatty acid composition (high oleic, high stearic, high oleic-high stearic, etc.).

#### Potential uses of the model

The model presented in this paper has been mainly targeted at simulating genetic effects on grain filling and composition dynamics. The identification of key genotypic parameters could guide future research on physiological processes and guide breeding programs. In addition, it is a promising tool to model the effects of biotic and abiotic factors on these dynamics. In this first version, the assimilates availability was estimated based on available data of canopy interception. However, the model could be linked to crop models capable of simulating it by considering different environmental input variables and calculating intermediate phenological or plant structure ones (e.g., Pereyra-Irujo and Aguirrezábal, 2007). In addition, using the present model as a platform and making the necessary modifications, it will be possible to explore the dynamics of other oilseed species (like soybean or rape), where enzymes and pathways are known to significantly differ from those in sunflower.

## Conclusion

In this work, a kinetic model of sunflower grain filling and fatty acids biosynthesis has been developed. The ability of the model to predict the experimental values was successfully evaluated and validated in different hybrids. The combination of sensitivity analysis and the genetic variability of parameters allowed minimizing the number of input parameters required to appropriately simulate the dynamics of grain filling and component accumulation in different hybrids. The growth model considered a simple effect of carbon source dynamics, maintenance requirements, and a simplified serial-parallel reaction system to describe the fatty acids biosynthetic pathway. The model developed represents a useful tool for future research to evaluate the effects of different factors on grain weight and composition, in a comprehensive and a quantitative way.

## Author contributions

In this work the skills from two different knowledge areas have been integrated. The experience of ID on kinetic modeling based on chemical reaction engineering basis was used to modelate experimental data from agricultural field. The experimental data have been obtained in INTA-BAlcarce by ME and LA during several years. ME provided the theoretical base and ID developed and programmed the kinetic model, aiming to represent the metabolic behavior of sunflower grain filling and fatty acids biosynthesis. All the authors wrote the manuscript.

### Conflict of interest statement

The authors declare that the research was conducted in the absence of any commercial or financial relationships that could be construed as a potential conflict of interest.

## Nomenclature

μ′: defined specific growth parameter [◦Cdaf^−1^]
C: carbohydrate equivalents [mgC]
C_*F*_: fatty acids synthesis intermediate [mg]
k_λ_: Lambert-Beer law adjusted constant [◦Cdaf^−1^]
k_max_: maximum specific rate [mg ◦Cdaf^−1^]
Ks: half saturation constant [mg mgW^−1^]
Kp: proportionality constant [dimensionless]
L: linoleic acid [mg]
m: maintenance coefficient [mgC ◦Cdaf^−1^ mgW^−1^]
pPAR: fraction of photosynthetic active radiation intercepted by the crop [unitless]
O: oleic acid [mg]
P: palmitic acid [mg]
r_i_: production/degradation rate of component i [mgi ◦Cdaf^−1^]
S: stearic acid [mg]
t: thermal time after flowering [◦Cd af]
W: dry weight of grain [mg]
W_0_: initial grain dry weight [mg]
W_max_: maximum theoretical grain dry weight [mg]
W_NO_: non-oil grain fraction dry weight [mg]
W_O_: oil grain fraction dry weight [mg]
Y_G_: actual growth yield coefficient or conversion efficiency [mg mgC^−1^]
Y_GNO_: actual non-oil fraction yield coefficient [mg mgC^−1^]
Y_GO_: actual oil fraction yield coefficient [mg mgC^−1^]

## Subscripts

− (minus sign): represents the inverse reaction
C: relative to substrate as carbohydrate equivalent
G: relative to growth
L: relative to linoleic acid
m: relative to maintenance
O: relative to oleic acid
P: relative to palmitic acid
S: relative to stearic acid
W: relative to grain weight
