# Supplementary material for: Kinetic Modeling of Sunflower Grain Filling and Fatty Acid Biosynthesis
Source: Front Plant Sci. 2016 May 6;7:586. doi: 10.3389/fpls.2016.00586 (PMC4863726; doi:10.3389/fpls.2016.00586)
Supplement: Supplementary file 1 [file Table1.docx]

**Table SM1: Kinetic parameters for ACA885, MG2 and DK3820 sunflower hybrids.** Within a row, values followed by the same letter are not significantly different (95% confidence interval)

| ***Parameter*** | ***ACA885*** | ***MG2*** | ***DK3820*** |
| --- | --- | --- | --- |
| **μ´ [°Cdaf^-1^]** | 0.0145 ± 0.0013 a | 0.0126± 0.0017 a | 0.018± 0.003 a |
| **W_0_ [mg]** | 0.4299± 0.1634a | 0.5862 ± 0.1906 a | 0.2400 ± 0.2273a |
| **W_max_ [mg]** | 34.5396 ± 0.9131a | 37.5079 ± 1.2389a | 39.35186 ± 1.67339a |
| **Y_G_ [mg.mg_C_^-1^]** | 0.671 ± 0.036 a | 0.950 ± 0.082 b | 0.638 ± 0.145 ab |
| **m [mg_C_.mg^-1^.°Cdaf^-1^]** | 4.31x10^-3^ ± 4.28x10^-4^ a | 4.25x10^-3^ ± 7.69x10^-4^ a | 2.43x10^-3^ ± 1.37x10^-4^ b |
| **Y_GO_ [mg.mg_C_^-1^]** | 0.303 ± 0.016 a | 0.406 ± 0.035 b | 0.208 ± 0.048 a |
| **v_maxP_ [mg.°Cdaf-1]** | 2.025x10^-4^± 6.769x10^-5^ a | 1.455x10^-4^ ± 6.815x10^-5^ a | 3.581x10^-4^ ± 9.157x10^-5^ a |
| **Ks_P_ [mg.mgW-1]** | 0.0139 ± 0.0022 a | 0.0106 ± 0.00137 a | 0.0755 ± 0.01855 b |
| **v_maxS_ [mg.°Cdaf-1]** | 7.783x10^-5^ ± 2.814x10^-5^ a | 1.6004x10^-4^ ± 1.107x10^-5^ b | 3.315x10^-4^ ± 9.216x10^-5^ b |
| **Ks_S_ [mg.mgW-1]** | 0.0214 ± 0.0029 b | 0.04537 ± 0.00114 a | 0.1336 ± 0.04891 a |
| **v_maxO_ [mg.°Cdaf-1]** | 3.157x10^-3^ ± 8.463x10^-4^ a | 3.410x10^-3^ ± 1.683x10^-4^ a | 3.912x10^-3^ ± 1.345x10^-4^ a |
| **Ks_O_ [mg.mgW-1]** | 0.0199 ± 0.0025 b | 0.0415 ± 0.0012 a | 0.03778 ± 0.00301 a |
| **v_maxL_ [mg.°Cdaf-1]** | 0.0197 ± 2.646x10^-3^ a | 0.0367 ± 5.049x10^-3^ b | 0.0440 ± 7.093x10^-3^ b |
| **Ks_L_ [mg.mgW-1]** | 0.0202 ± 0.0322 ab | 0.0595 ± 0.0017 a | 0.0423 ± 0.0052 b |
| **v_max-L_ [mg.°Cdaf-1]** | 0.0190 ± 6.837x10^-4^ a | 0.0291 ± 4.032x10^-4^ b | 0.0349 ± 6.121x10^-4^ c |
| **Ks_-L_ [mg.mgW-1]** | 0.0159 ± 0.0022 a | 0.0391 ± 0.0021 b | 0.0723 ± 0.0036 c |
