# Supplementary material for: Kinetic Modeling of Sunflower Grain Filling and Fatty Acid Biosynthesis
Source: Front Plant Sci. 2016 May 6;7:586. doi: 10.3389/fpls.2016.00586 (PMC4863726; doi:10.3389/fpls.2016.00586)
Supplement: Supplementary file 2 [file DataSheet1.DOCX]

SUPPLEMENTARY MATERIAL

Kinetic modeling of sunflower grain filling and fatty acid biosynthesis

Appendix: Expanded model development for grain growth and maintenance and grain filling

Here we present a more detailed description of some model sections with the aim of explaining the rationale of many equations and to promote its use and reproducibility. Focus is made on equations supporting grain growth, substrate consumption and grain filling

**Grain growth**

The mass balance for a grain growing in batch conditions can be written as

$\boldsymbol{0=0+}\frac{\boldsymbol{dW}}{\boldsymbol{dt}}\boldsymbol{-}\boldsymbol{r}_{\boldsymbol{W}}$ (SM1)

where W is the dry weight of an individual grain and *r_W_* represents the growth rate. One of the most widely used expressions for growth rate in biological systems is the Monod equation:

$\boldsymbol{r}_{\boldsymbol{W}}\boldsymbol{=}\frac{\boldsymbol{\mu}_{\boldsymbol{max}}\boldsymbol{.C}}{\boldsymbol{Ks+C}}\boldsymbol{.W}$ (SM2)

where C represents the substrate for growth expressed as carbohydrate equivalents (Vertregt and Penning de Vries, 1987; Echarte *et al.*, 2012), and μ_max_ and Ks are the maximum specific growth rate and the half saturation constant, respectively. Since the substrate is rapidly consumed once it enters the grain, we can assume C inside the grain is always low and thus Equation (SM2) can be simplified to:

$\boldsymbol{r}_{\boldsymbol{W}}\boldsymbol{=}\frac{\boldsymbol{\mu}_{\boldsymbol{max}}}{\boldsymbol{Ks}}\boldsymbol{C.W}$ (SM3)

Defining C* as the theoretical amount of substrate needed to produce the potential grain weight (W_max_) in the absence of maintenance costs, the net growth yield (Y_W/C_) can be expressed as:

$\boldsymbol{Y}_{\boldsymbol{W/C}}\boldsymbol{=}\frac{\boldsymbol{W}_{\boldsymbol{max}}\boldsymbol{-}\boldsymbol{W}_{\boldsymbol{0}}}{\boldsymbol{C}^{\boldsymbol{*}}}$ (SM4)

where W_0_ is the initial amount of W at which the model starts to work. Substrate can be expressed as:

$\boldsymbol{C=}\boldsymbol{C}^{\boldsymbol{*}}\boldsymbol{-}\frac{\boldsymbol{W-}\boldsymbol{W}_{\boldsymbol{0}}}{\boldsymbol{Y}_{\boldsymbol{w/c}}}\boldsymbol{=}\frac{\boldsymbol{W}_{\boldsymbol{max}}\boldsymbol{-}\boldsymbol{W}_{\boldsymbol{0}}}{\boldsymbol{Y}_{\boldsymbol{w/c}}}\boldsymbol{-}\frac{\boldsymbol{W-}\boldsymbol{W}_{\boldsymbol{0}}}{\boldsymbol{Y}_{\boldsymbol{w/c}}}\boldsymbol{=}\frac{\boldsymbol{W}_{\boldsymbol{max}}\boldsymbol{-W}}{\boldsymbol{Y}_{\boldsymbol{w/c}}}$ (SM5)

And therefore the growth rate can be written as

$\boldsymbol{r}_{\boldsymbol{W}}\boldsymbol{=}\frac{\boldsymbol{\mu}_{\boldsymbol{max}}}{\boldsymbol{Ks}}\boldsymbol{.}\frac{\boldsymbol{W}_{\boldsymbol{max}}\boldsymbol{-W}}{\boldsymbol{Y}_{\boldsymbol{w/c}}}\boldsymbol{.W}$ (SM6)

Equation (SM6) can be found on the body manuscript as Equation 2.

$\boldsymbol{r}_{\boldsymbol{W}}\boldsymbol{=\mu´.}\frac{\boldsymbol{W}_{\boldsymbol{max}}\boldsymbol{-W}}{\boldsymbol{W}_{\boldsymbol{max}}}\boldsymbol{.W}$ **(SM 7)**

After integrating Equation (SM7) with the initial condition W=W_0_, the logistic Equation (SM8) is obtained:

$\boldsymbol{W=}\frac{\boldsymbol{W}_{\boldsymbol{0}}\boldsymbol{.}\boldsymbol{e}^{\boldsymbol{\mu´.t}}}{\boldsymbol{1-}\frac{\boldsymbol{W}_{\boldsymbol{0}}}{\boldsymbol{W}_{\boldsymbol{max}}}\left( \boldsymbol{1-}\boldsymbol{e}^{\boldsymbol{\mu´.t}} \right)}$ (SM8)

Monod expressed Equation (SM8) - Equation 1 in the manuscript main body- as a particular case of Equation SM6, where μ´can be written as:

$\mu´=\frac{\frac{\mu_{max}}{Ks}.W_{max}}{Y_{W/C}}$ (SM9)

In Equation SM9 μ´ represents the specific grain growth rate (i.e the biomass production rate per unit of biomass).

**Carbohydrate equivalent mass balance.**

Considering that the grain continuously receives the substrate assimilated by the mother plant, the C that enters into the grain per time unit (F_C_) is equal to the consumption rate (*r_C_*) plus the accumulation rate. Furthermore, since the substrate reacts as soon as it enters the grain, the accumulation rate can be neglected

$\boldsymbol{F}_{\boldsymbol{C}}\boldsymbol{=}\boldsymbol{r}_{\boldsymbol{C}}\boldsymbol{+acumulation}$ (SM10)

$\boldsymbol{F}_{\boldsymbol{C}}\boldsymbol{=}\boldsymbol{r}_{\boldsymbol{C}}$ (SM10)

However, not all the substrate reaching the grain is used for growth. Part of C is consumed in maintenance, providing the energy needed for diverse processes that do not result in a net increase of dry weight. The Pirt´s maintenance equation (Pirt, 1975) allows us to take into account these maintenance processes:

$\boldsymbol{r}_{\boldsymbol{C}}\boldsymbol{=}\frac{\boldsymbol{r}_{\boldsymbol{W}}}{\boldsymbol{Y}_{\boldsymbol{G}}}\boldsymbol{+m.W}$ (SM11)

being *m* the maintenance coefficient and Y_G_ the actual growth yield coefficient.

As the first term of Equation (SM11) represents the substrate used for grain growth and the second one the substrate used for maintenance purposes, *r_C_* can be split into the rate of substrate consumption for growth (*r_CG_*) and the rate of substrate consumption for maintenance (*r_Cm_*):

$\boldsymbol{r}_{\boldsymbol{CG}}\boldsymbol{=}\frac{\boldsymbol{r}_{\boldsymbol{W}}}{\boldsymbol{Y}_{\boldsymbol{G}}}$ (SM12)

$\boldsymbol{r}_{\boldsymbol{Cm}}\boldsymbol{=m.W}$ (SM13)

Considering dry weight W is composed by oil (W_O_) and non-oil (W_NO_) fractions, the production rates can be written as:

$\boldsymbol{r}_{\boldsymbol{WO}}\boldsymbol{=}\boldsymbol{Y}_{\boldsymbol{GO}}\boldsymbol{.}\boldsymbol{r}_{\boldsymbol{CG}}$ (SM14)

$\boldsymbol{r}_{\boldsymbol{WNO}}\boldsymbol{=}\boldsymbol{Y}_{\boldsymbol{GNO}}\boldsymbol{.}\boldsymbol{r}_{\boldsymbol{CG}}$ (SM15)

where *Y_GO_* and *Y_GNO_* are the actual oil and non-oil fraction yield coefficients, respectively. Note that since *r_WO_* plus *r_WNO_* (growth rate of oil and non-oil components, respectively) must be equal to *r_W_*, *Y_G_* results equal to *Y_GO_* plus *Y_GNO_*. Furthermore, *r_CG_* can be fractionated into the rates of substrate consumption for non-oil fraction (*r_CNO_*) and oil fraction (*r_CO_*):

$\boldsymbol{r}_{\boldsymbol{CNO}}\boldsymbol{=}\frac{\boldsymbol{Y}_{\boldsymbol{GNO}}}{\boldsymbol{Y}_{\boldsymbol{G}}}\boldsymbol{.}\boldsymbol{r}_{\boldsymbol{CG}}$ (SM16)

$\boldsymbol{r}_{\boldsymbol{CO}}\boldsymbol{=}\frac{\boldsymbol{Y}_{\boldsymbol{GO}}}{\boldsymbol{Y}_{\boldsymbol{G}}}\boldsymbol{.}\boldsymbol{r}_{\boldsymbol{CG}}$ (SM17)

**Grain filling dynamics**

Changes in the contribution of C from the mother plant to grain filling where estimated by considering changes in photosynthetically active radiation interception as stated in section 2.1.3 of the manuscript main body:

$\boldsymbol{F}_{\boldsymbol{C}}\boldsymbol{=}\boldsymbol{r}_{\boldsymbol{C}}\boldsymbol{.p}_{\boldsymbol{PAR}}$ (SM18)

and the theoretical accumulation of carbohydrate equivalents, i.e. the cumulative amount of substrate that enters the grain results:

$\frac{\boldsymbol{dC}}{\boldsymbol{dt}}\boldsymbol{=}\left( \frac{\boldsymbol{r}_{\boldsymbol{W}}}{\boldsymbol{Y}_{\boldsymbol{G}}}\boldsymbol{+m.W} \right)\boldsymbol{.p}_{\boldsymbol{PAR}}$ (SM19)

Then, the substrate used for growth and maintenance can be individually predicted by the following equations:

$\frac{\boldsymbol{d}\boldsymbol{C}_{\boldsymbol{NO}}}{\boldsymbol{dt}}\boldsymbol{=}\boldsymbol{r}_{\boldsymbol{CNO}}\boldsymbol{.p}_{\boldsymbol{PAR}}\boldsymbol{=}\frac{\boldsymbol{Y}_{\boldsymbol{GNO}}}{\boldsymbol{Y}_{\boldsymbol{G}}}\boldsymbol{r}_{\boldsymbol{CG}}\boldsymbol{.p}_{\boldsymbol{PAR}}$ (SM20)

$\frac{\boldsymbol{d}\boldsymbol{C}_{\boldsymbol{O}}}{\boldsymbol{dt}}\boldsymbol{=}\boldsymbol{r}_{\boldsymbol{CO}}\boldsymbol{.p}_{\boldsymbol{PAR}}\boldsymbol{=}\frac{\boldsymbol{Y}_{\boldsymbol{GO}}}{\boldsymbol{Y}_{\boldsymbol{G}}}\boldsymbol{r}_{\boldsymbol{CG}}\boldsymbol{.p}_{\boldsymbol{PAR}}$ (SM21)

$\frac{\boldsymbol{d}\boldsymbol{C}_{\boldsymbol{m}}}{\boldsymbol{dt}}\boldsymbol{=}\boldsymbol{r}_{\boldsymbol{Cm}}\boldsymbol{.p}_{\boldsymbol{PAR}}\boldsymbol{=m.W}\boldsymbol{.p}_{\boldsymbol{PAR}}$ (SM22)

And the production of W and its fractions can be calculated as:

$\frac{\boldsymbol{dW}}{\boldsymbol{dt}}\boldsymbol{=}\boldsymbol{Y}_{\boldsymbol{G}}\boldsymbol{.r}_{\boldsymbol{CG}}\boldsymbol{.p}_{\boldsymbol{PAR}}\boldsymbol{=}\boldsymbol{r}_{\boldsymbol{W}}\boldsymbol{.p}_{\boldsymbol{PAR}}$(SM23)

$\frac{\boldsymbol{d}\boldsymbol{W}_{\boldsymbol{NO}}}{\boldsymbol{dt}}\boldsymbol{=}\boldsymbol{Y}_{\boldsymbol{GNO}}\boldsymbol{.r}_{\boldsymbol{CG}}\boldsymbol{.p}_{\boldsymbol{PAR}}\boldsymbol{=}\boldsymbol{r}_{\boldsymbol{WNO}}\boldsymbol{.p}_{\boldsymbol{PAR}}$ (SM24)

$\frac{\boldsymbol{d}\boldsymbol{W}_{\boldsymbol{O}}}{\boldsymbol{dt}}\boldsymbol{=}\boldsymbol{Y}_{\boldsymbol{GO}}\boldsymbol{.r}_{\boldsymbol{CG}}\boldsymbol{.p}_{\boldsymbol{PAR}}\boldsymbol{=}\boldsymbol{r}_{\boldsymbol{WO}}\boldsymbol{.p}_{\boldsymbol{PAR}}$ (SM25)

# Reference are included in the manuscript main body
